# Anger as a Bridge: Understanding How Climate Change Anxiety Shapes Well-Being

**DOI:** 10.1177/24705470261468139

**Published:** 2026-08-08

**Authors:** Mariana Pinho, Enrickson Varsori

**Affiliations:** 1 Centre for Environmental and Marine Studies, 56062 University of Aveiro, Aveiro, Portugal; 2 School of Psychology and Life Sciences, Lusófona University, Lisboa, Portugal; 3 CECS - Communication and Society Research Centre, 56059 University of Minho, Braga, Portugal

**Keywords:** climate change anxiety, anxiety, well-being, mental health, anger

## Abstract

**Background:**

Climate change anxiety is increasingly recognized as a mental health concern, with emerging evidence linking it to poorer well-being and highlighting the need to clarify its cognitive-emotional and functional dimensions.

**Methods:**

The current research explored the relationship between climate anxiety and mental health outcomes, using a large nationally representative sample.

**Results:**

The results revealed that climate change anxiety was positively related to negative affect and anger. Climate anxiety cognitive impairment was positively related to positive affect and life satisfaction. However, climate anxiety functional impairment showed a different pattern, being negatively associated with life satisfaction at the bivariate level but no longer remaining a significant predictor once generalized anxiety state and trait were included in the model. Climate change anxiety showed moderate positive correlations with generalized anxiety, both state and trait. Finally, in exploratory cross-sectional mediation models, anger statistically accounted for part of the association between climate change anxiety and both negative affect and life satisfaction, but not positive affect.

**Conclusion:**

This study contributes to a more nuanced understanding of the psychological processes involved in climate anxiety and its relation with subjective well-being.

## Introduction

An increasing number of studies have examined the effects of climate change on mental health (e.g., Refs. 1 and 2). Specifically, recent research has shown the negative impact of climate change anxiety on well-being (e.g., Refs. 3 and 4). This is particularly relevant because climate anxiety may involve both cognitive-emotional and functional impairment, making it important to clarify how it relates to broader mental health constructs and to different dimensions of well-being.^1^

The increasing frequency and intensity of climate-related events, such as wildfires, floods, and heatwaves, have posed physical threats and precipitated psychological distress among affected populations. Importantly, climate anxiety may also arise from indirect exposure and awareness of climate change, even among individuals who have not personally experienced climate-related disasters, suggesting that its psychological impact extends beyond direct trauma exposure.^1^ Climate change anxiety refers to the chronic fear of environmental doom resulting from the awareness of climate change and its potential impacts.^5^ This phenomenon has garnered attention due to its implications for mental health, particularly among younger individuals and communities directly impacted by climate events.

Recent studies have established a positive correlation between climate change anxiety and various mental health issues and psychological distress (e.g., depression, anxiety disorders, stress, post-traumatic stress disorder).^6-8^ Moreover, vulnerable groups have shown increased prevalence of suicidal ideation and suicide plans following severe climate events.^6^ Despite the increasing focus on climate anxiety (e.g. Refs. 1 and 9), research has shown mixed results , with studies linking it to both negative^1,10^ and positive emotions.^11-13^ This suggests that climate anxiety should not be understood only as a marker of psychopathology, but also as a complex emotional response that may coexist with concern, engagement, or other affective reactions to climate change. Therefore, there is still limited understanding of its effects on mental health, as previous studies have mainly examined how alterations in the physical environment impact individuals’ emotional experiences (e.g., Ref. 14). The current research aims to enhance our understanding of the relationship between climate change anxiety and mental health outcomes, using a large nationally representative sample and highlighting the psychological distress associated with climate change.

### Climate Change Anxiety and Well-Being

Several factors contribute to the development and intensity of climate change anxiety. These include direct exposure to climate-related disasters, media consumption, and socio-political contexts. Namely, higher direct exposure to climate disasters (e.g., Ref. 15), media consumption (e.g., Ref. 16) and the perception of inadequate government response (e.g., Ref. 17) are related to higher levels of climate change anxiety. Thus, climate anxiety appears to be shaped not only by exposure to climate-related events but also by how individuals interpret, encounter, and make sense of climate change through social and informational contexts. It has also been documented that people who directly experience climate disasters have been shown to experience mood disorders (e.g. Refs. 18-21), including exhibiting symptoms of post-traumatic stress disorder (PTSD) (e.g. Refs. 21-23) and anxiety disorders (e.g. Refs. 8, 19 and 21). Additionally, demographic factors such as age, gender, and educational background influence the levels of climate change anxiety. Studies have shown that younger individuals, women and people with higher education degrees report higher levels of concern about climate change and its implications for their future.^1,5,24^

An increasing number of studies have also examined the extent to which climate anxiety affects subjective well-being and mental health. Previous research has shown that climate distress or worry has been associated with psychological distress (e.g., Refs. 25-27), negatively linked with mental well-being globally^3,28^ and to predict emotional and cognitive dysfunction.^1^ This effect was found even after one year.^29^ While a few studies found positive correlations between climate anxiety and symptoms of major depressive disorder,^1,30^ others did not find a significant relationship between climate anxiety and depression symptoms.^13^ These mixed findings suggest that climate anxiety may overlap with established indicators of psychological distress while also retaining features that are specific to climate-related threat, uncertainty, and future-oriented concern.

Furthermore, climate anxiety has been strongly associated with anxiety symptoms and generalized anxiety disorder.^1,13,30,31^ However, because climate anxiety is anchored in a specific collective and environmental threat, it remains important to examine whether its associations with well-being persist when generalized anxiety is considered.

Among the emotional responses associated with climate change, anger may be particularly relevant for understanding how climate anxiety relates to well-being. Climate-related anger may emerge when concern about climate change is accompanied by perceptions of injustice, frustration, or inadequate collective and political responses. Previous research has suggested that anger and frustration are important emotional responses to climate change and should be examined alongside anxiety, sadness, fear, and other negative emotions.^1,13,32^

Overall, climate change anxiety represents a significant and growing mental health concern that warrants immediate attention. The psychological impacts of climate anxiety are profound and multifaceted, affecting individuals’ well-being. However, further research is needed to clarify whether climate anxiety is associated with subjective well-being beyond generalized anxiety and to examine whether specific emotional responses, such as anger, are statistically involved in these associations. Consequently, by exploring the associations between climate anxiety and well-being and by examining whether anger statistically accounts for part of these associations, the present study aims to contribute to a greater understanding of climate anxiety in relation to mental health outcomes and to better equip individuals to cope with the challenges posed by climate change.

## Method

### Participants and Procedure

Data were collected from 3,300 adults (48% men and 52% women), recruited via an online questionnaire distributed to members of Qdata’s panel, which comprises 860,000 individuals. Qdata is one of Portugal’s leading survey firms, with a nationally representative panel. The sample size was determined based on a 2% margin of error and a 95% confidence level, reflecting the overall Portuguese population. Stratified random sampling was employed to ensure that all demographic groups were represented in the same proportions as in the national population. Participants ranged in age from 18 to 74 years (*M* = 46.29, *SD* = 13.96), with most living in urban areas (64.4%), being married or in a civil partnership (59%), and having children (62.3%). Seventy-two per cent were employed, more than half held a higher education degree (55.4%), and almost half earned a monthly household income between €891 and €2083 (49%). Participation was entirely voluntary and anonymous. On average, completing the questionnaire took 15 minutes. Participants were thanked and debriefed at the end of the questionnaire.

### Measures

#### Climate Change Anxiety

Climate anxiety as a psychological response to climate change was measured using^1^ 13-item instrument. The measure is composed of two subscales: a) cognitive-emotional impairment (e.g., “*Thinking about climate change makes it difficult for me to sleep*”) and b) functional impairment (e.g., “*I have problems balancing my concerns about sustainability with the needs of my family*”). Responses were indicated on a 5-point scale ranging from 1= *Never* to 5 = *Almost always*. The respondent’s average score on each dimension was computed. The average of all 13 items was also calculated to obtain a total climate change anxiety score. Cronbach’s alpha for the overall climate change anxiety scale was 0.94, for the cognitive-emotional impairment subscale was 0.91 and the functional impairment subscale was 0.89.

#### Subjective Well-Being

Subjective well-being was assessed using the *Positive and Negative Affect Scale* (PANAS) (Ref. 33, Portuguese version^34^). The scale contains two dimensions: Positive Affect (PA) reflects the extent to which a person feels enthusiastic, active, and alert (e.g. “*Interested*”); and Negative Affect (NA) reflects subjective distress and displeasure (e.g. *“Distressed*”). The measure had 20 items and responses were recorded on a scale from 1 = *Very Slightly/Not at All* to 5 = *Extremely*. The items for each dimension were averaged separately to create the participants’ scores on two scales. On the PA sub-dimension, higher scores represented higher levels of positive affect, while higher scores on the NA sub-dimension represented higher levels of negative affect. Cronbach’s alpha was 0.91 for each of these scales. Additionally, anger and calm were added as two additional emotions, as previous literature revealed important connections with climate change anxiety (e.g., Refs. 13 and 32). These emotions were assessed as additional single-item indicators and analyzed separately from the PANAS positive and negative affect subscales.

#### Life Satisfaction

Life satisfaction was measured with the *Satisfaction with Life Scale* (SWLS), a five-item measure developed by Ref. 35 and validated for the Portuguese population by Ref. 36, to assess satisfaction with the respondent’s life as a whole. Subjective well-being is defined as cognitive and affective evaluations of one’s life.^37^ This measure relates to the cognitive evaluative component of subjective well-being. Participants answered on a 7-point scale ranging from 1 = *Strongly Disagree* to 7 = *Strongly Agree* to indicate the extent to which they agreed with each statement (e.g. *“In most ways my life is close to my ideal*”). The average score for the five items was computed to measure participants’ life satisfaction. Higher scores on this measure reflected higher levels of satisfaction. Cronbach’s alpha for this measure was 0.90.

#### Cognitive and Somatic Anxiety

Cognitive and somatic symptoms of state and trait anxiety were measured using the 42-item self-report instrument, State-Trait Inventory for Cognitive and Somatic Anxiety (STICSA -^38^; Portuguese validated version by Ref. 39. The instrument is divided into two scales, one evaluating anxiety in its state dimension (“*How do you feel right now*”; STICSA-State) and the other evaluating its trait dimension (“*How often this is true for you*?”; STICSA-Trait), with both scales using the same 21 items. Responses were recorded on a 4-point scale that ranged from 1 = *Not at all* to 4 = *Very much*. Cronbach’s alpha for the two scales was 0.94 and 0.95, respectively.

#### Socio-Demographic Variables

Participants indicated their age, gender, occupation, level of education, and marital status. Participants also reported their individual monthly income on a seven-point scale ranging from 1 (*less than €590*) to 7 (*more than €6720*).

## Results

### Preliminary Analysis

Variance Inflation Factor values were below the conventional threshold of 5, suggesting that multicollinearity was not severe in the regression models^40^ (see Table 1).

**Table 1.** Variance Inflation Factor (VIF)

​	VIF
Climate anxiety cognitive-emotional impairment	2.79
Climate anxiety functional impairment	2.76
Generalized anxiety state	4.96
Generalized anxiety trait	4.84

Means, standard deviations, and Pearson correlations among climate change anxiety, subjective well-being, and sociodemographic characteristics are presented in Table 2.

**Table 2.** Means, Standard Deviations, and Correlations Among Study Measures

*Variables*	1	2	3	4	5	6	7	8	9
1. Climate Anxiety Cognitive Impairment	*--*	​	​	​	​	​	​	​	​
2. Climate Anxiety Functional Impairment	.80***	*--*	​	​	​	​	​	​	​
3. Negative Affect	.46***	.45***	--	​	​	​	​	​	​
4. Anger	.29***	.28***	.69***	--	​	​	​	​	​
5. Positive Affect	.20***	.14***	.11***	.03	--	​	​	​	​
6. Calm	-.02	-.04*	-.26***	-.23***	.54***	--	​	​	​
7. Life Satisfaction	.04*	-.02	-.23***	-.23***	.36***	.34***	--	​	​
8. STICSA-State	.43***	.40***	.65***	.47***	.01	-.23***	-.29***	--	​
9. STICSA-Trait	.39***	.37***	.64***	.47***	-.01	-.24***	-.27***	.89***	--
*M*	1.81	1.68	2.00	1.91	2.87	3.10	4.06	1.71	1.69
*SD*	0.70	0.73	0.77	1.09	0.78	1.04	1.30	0.56	0.59

*Note.* Higher scores on all measures reflect higher levels of the construct. *p < .05; **p < .01; ***p < .001.

The results show that climate change anxiety was positively related to negative affect (*r* = .46, *r* = .45) and anger (*r* = .29, *r* = .28, for cognitive and functional impairment, respectively), suggesting that the higher levels of climate anxiety participants experienced, the greater levels of negative affect and anger they reported. On the other hand, functional impairment due to climate change anxiety was negatively related to calm (*r* = -.04). Climate anxiety was also positively related to positive affect (*r* = .20, *r* = .14, for cognitive and functional impairment, respectively).

Climate change anxiety had moderate positive associations with generalized anxiety, state and trait (*r* = .43, *r* = .39, *r* = .40, *r* = .37, for cognitive and functional impairment, respectively), indicating that climate anxiety and generalized anxiety were related, although not fully overlapping, at the bivariate level.

Generalized anxiety, state and trait, were also positively related to negative affect (*r* = .65, *r* = .64, respectively), anger (*r* = .47), while negatively related to calm (*r* = -.23, *r* = -.24) and life satisfaction (*r* = -.29, *r* = -.27, respectively).

### Climate Change Anxiety and Subjective Well-Being

To explore the role of climate change anxiety, generalized anxiety state and trait on subjective well-being, a set of multiple regression analyses was conducted (Table 3). The cognitive and functional impairment dimensions of climate anxiety were entered simultaneously in Model 1, followed by generalized anxiety state in Model 2 and generalized anxiety trait in Model 3. In the final model predicting negative affect, both climate anxiety cognitive impairment (β = .11, *p* < .001) and functional impairment (β = .14, *p* < .001) remained significant positive predictors. Generalized anxiety state (β = .26, *p* < .001) and trait (β = .32, *p* < .001) were also significant positive predictors of negative affect.

**Table 3.** Multiple Regression Analyses Predicting Negative Affect, Positive Affect and Life Satisfaction From Climate Anxiety, Cognitive, Somatic Anxiety State and Trait

Model	Negative affect			Positive affect			Life satisfaction		
	1	2	3	1	2	3	1	2	3
Climate Anxiety Cognitive Impairment	.25***	.11***	.11***	.15***	.17***	.17***	.11***	.20***	.20***
Climate Anxiety Functional Impairment	.25***	.15***	.14***	.03	.05	.05	-.10***	-.03	-.03
STICSA-State	--	.53***	.26***	--	-.10***	-.08*	--	-.37***	-.30***
STICSA-Trait	--	--	.32***	--	--	-.02	--	--	-.08*
*R* ^*2*^	.22***	.46***	.48***	.03***	.04***	.04***	.01***	.11***	.12***
*F*(4, 3244)	​	734.74***		​	​	31.46***	​	110.30***	

*Note.* Standardized beta coefficients are reported. Tests of significance were two-tailed. *p < .05; ***p < .001.

For positive affect, climate anxiety cognitive impairment remained a significant positive predictor in the final model (β = .17, *p* < .001), whereas functional impairment was not significant. Generalized anxiety state was a significant negative predictor of positive affect (β = -.08, *p* < .05), while generalized anxiety trait was not significant. For life satisfaction, climate anxiety cognitive impairment was a significant positive predictor in the final model (β = .20, *p* < .001), whereas functional impairment was not significant after generalized anxiety state and trait were included. Generalized anxiety state (β = -.30, *p* < .001) and trait (β = -.08, *p* < .05) were significant negative predictors of life satisfaction.

To explore whether anger statistically accounted for part of the association between climate change anxiety and subjective well-being, methods developed by Ref. 41 were followed. These analyses were conducted using the PROCESS program (Model 4; Ref. 42) with bias-corrected bootstrap estimates and 95% confidence intervals. Table 4 illustrates the results of the mediation analyses. Separate mediation models were estimated for the cognitive-emotional and functional impairment dimensions of climate anxiety. These results indicate that climate change anxiety had an indirect effect on negative affect and life satisfaction. This effect was mediated by anger, as indicated by bootstrap confidence intervals entirely above zero for negative affect (95% CI [.164, .215], [.155, .203] for cognitive and functional impairment) and entirely below zero for life satisfaction (95% CI [-.165, -.114], [-.152, -.104] for cognitive and functional impairment). The indirect effects on positive affect were not statistically significant, as the confidence intervals included zero. These results suggest that anger statistically accounted for part of the associations between climate change anxiety and negative affect and life satisfaction, but not positive affect.

**Table 4.** Bias-Corrected Bootstrap Estimates for Climate Anxiety With Mediation by Anger

​	Negative affect			Positive affect			Life satisfaction		
	Estimate	95% CI		Estimate	95% CI		Estimate	95% CI	
		Lower	Upper		Lower	Upper		Lower	Upper
*Climate Anxiety Cognitive Impairment*									
Direct effect	.309***	.284	.335	.235***	.196	.274	.219***	.155	.282
Indirect effect	.189***	.164	.215	-.011	-.022	.001	-.138***	-.165	-.114
*Climate Anxiety Functional Impairment*									
Direct effect	.283***	.258	.308	.161***	.124	.199	.099***	.037	.160
Indirect effect	.178***	.155	.203	-.010	-.021	.001	-.127***	-.152	-.104

*Note.* CI = confidence interval. ***p < .001.

## Discussion

The current study explored the relationship between climate change anxiety and mental health outcomes, using a large nationally representative sample and highlighting the psychological distress associated with climate change. Echoing recent research, the results revealed that both cognitive and functional impairment dimensions of climate change anxiety were positively related to negative affect (e.g., Refs. 1 and 13). The same pattern of results was found for anger, consistent with previous research.^13^ Functional impairment due to climate change anxiety was negatively related to calm, extending previous findings on the link between climate anxiety and mental well-being. Interestingly, climate anxiety cognitive impairment was positively related to positive affect and life satisfaction, confirming previous findings.^11-13,29,43^ However, climate anxiety functional impairment showed a different pattern, being negatively associated with life satisfaction at the bivariate level but no longer remaining a significant predictor once generalized anxiety state and trait were included in the model.

The positive associations between climate anxiety cognitive-emotional impairment and positive affect and life satisfaction are consistent with previous work suggesting that climate-related anxiety, concern, and negative emotions may coexist with engagement, motivation, or mixed well-being outcomes.^11-13,29^ However, it is important to note that the cognitive-emotional subscale assesses impairment rather than adaptive engagement or positive functioning. One possible explanation is that this pattern reflects measurement complexity, shared variance with broader climate concern, salience, or engagement, or other unmeasured variables, rather than indicating that impairment is beneficial. Nevertheless, the distinction between cognitive and functional impairment is relevant. Whereas cognitive impairment was positively associated with positive affect and life satisfaction, functional impairment showed weaker or less consistent associations with these outcomes once generalized anxiety was considered. This pattern suggests that the associations between climate anxiety and well-being may vary depending on the dimension examined. However, given the high correlation between the cognitive-emotional and functional impairment subscales, these differences should be interpreted cautiously.

Climate change anxiety showed moderate positive correlations with generalized anxiety, both state and trait, suggesting overlap between climate anxiety and generalized anxiety. Given the strong role of generalized anxiety in the regression models, these associations should be interpreted with this broader emotional vulnerability in mind. At the same time, dimensions of climate anxiety remained associated with some subjective well-being outcomes after generalized anxiety state and trait were included. Additionally, this is consistent with findings observed worldwide.^1,13,30,31^

Finally, the exploratory mediation analyses suggested that anger statistically accounted for part of the associations between climate anxiety and both negative affect and life satisfaction, but not positive affect. This pattern was observed for both cognitive and functional impairment dimensions of climate anxiety, suggesting that anger may be an important emotional correlate in the link between climate-related impairment and subjective well-being. However, because the present data are cross-sectional, these findings should be interpreted as statistical mediation rather than evidence of causal pathways.

Although experiencing climate anxiety may not, in itself, meet the criteria for a clinical disorder, it can nonetheless co-occur with or intensify psychological distress in some individuals. Given documented associations between climate anxiety and mental health indicators, their co-occurrence is likely. Considering this, it is essential that mental health professionals possess adequate climate literacy to effectively engage with climate anxiety in therapeutic contexts.^30,44^ This study contributes to a more nuanced understanding of the psychological processes involved in climate anxiety and its dual effects on subjective well-being. Results can inform the development of more effective mental health interventions and public policy strategies that address the emotional complexity of climate-related distress.

By examining climate anxiety within a different cultural context and analyzing a nationally representative sample, this study extends existing literature by demonstrating that the psychological impacts of climate anxiety are not confined to English-speaking populations. This cross-cultural evidence supports the growing recognition that climate anxiety is a global phenomenon with complex associations with subjective well-being, reinforcing the need for culturally sensitive approaches in both research and intervention strategies.

Climate anxiety and anger in Portugal may be shaped by national experiences of climate-related risks, including heatwaves, drought, and wildfire events, as well as by media coverage, public debate, political trust, and perceptions of institutional responsiveness.^17,45,46^ In this context, climate-related anger may reflect not only individual distress but also frustration with perceived collective or governmental inaction, particularly given the salience of wildfire risk, youth climate litigation and concerns about the adequacy of national and international climate policy.^13,17,47^ At the same time, cultural norms regarding emotional expression, civic responsibility, and environmental concern may influence how individuals report climate anxiety and anger.^48^

### Limitations and Future Research

The present study relied on self-reporting measures, which represent a methodological issue as single-source self-reports could be affected by social desirability.^49^ Future research would benefit from incorporating diverse measurement methods, including experimental designs, to assess climate change anxiety. In addition, anger was assessed as an additional single-item indicator rather than as a multidimensional measure of climate-related anger. Although this allowed anger to be included in a large-scale survey, single-item measures do not allow internal consistency to be estimated and may not capture important dimensions of anger, such as intensity, duration, target, perceived injustice, or action tendency. Because anger was central to the exploratory mediation models, these findings should be interpreted cautiously. Future studies should examine whether more specific measures of climate anger produce similar statistical indirect-effect patterns. In addition, although the Climate Change Anxiety Scale has been previously validated, the present study did not conduct additional factor-analytic analyses. Given the high correlation between the cognitive-emotional and functional impairment subscales, future research should further examine the factorial structure and discriminant validity of these dimensions in Portuguese samples.

Also noteworthy is the cross-sectional nature of this study, which prevents the extraction of causal conclusions with confidence. Future research should collect longitudinal data to explore how socio-demographic and social psychological characteristics, climate change anxiety, anger and well-being are related to each other over time. Longitudinal research could also assess the impact of sustained, long-term exposure to climate-related stressors on mental health. This is particularly important because the present mediation analyses should be interpreted as statistical mediation rather than evidence of causal pathways.

Regarding broader domains of life and health, although the present findings have implications for understanding how climate anxiety may relate to broader domains of life and health, this study focused specifically on subjective well-being outcomes, namely positive affect, negative affect, and life satisfaction. It did not directly assess daily functioning, social relationships, occupational performance, health behaviors, or clinical symptoms. Future research should examine whether the distinction between cognitive and functional impairment in climate anxiety has implications for these broader outcomes.

In conclusion, this study reinforces the expanding body of research demonstrating the meaningful role of climate anxiety in shaping various dimensions of well-being.

## Data Availability

Due to the nature of the research, supporting data is not available.*
